# Rapid high-yield expression of a candidate influenza vaccine based on the ectodomain of M2 protein linked to flagellin in plants using viral vectors

**DOI:** 10.1186/s12896-015-0164-6

**Published:** 2015-05-29

**Authors:** Eugenia S. Mardanova, Roman Y. Kotlyarov, Victor V. Kuprianov, Liudmila A. Stepanova, Liudmila M. Tsybalova, George P. Lomonosoff, Nikolai V. Ravin

**Affiliations:** Centre ‘Bioengineering’, Russian Academy of Sciences, Prosp. 60-letya Oktyabrya, bld 7-1, 117312 Moscow, Russia; Research Institute of Influenza, Russian Federation Ministry of Health, 15/17 Prof. Popova str., 197376 St. Petersburg, Russia; Department of Biological Chemistry, John Innes Centre, Norwich Research Park, NR4 7UH Norwich, UK

**Keywords:** Viral vector, Potato virus X, M2 protein, Flagellin, Influenza, Vaccine

## Abstract

**Background:**

The extracellular domain of matrix protein 2 (M2e) of influenza A virus is a promising target for the development of a universal vaccine against influenza because M2e sequences are highly conserved among human influenza A strains. However, native M2e is poorly immunogenic, but its immunogenicity can be increased by delivery in combination with adjuvants or carrier particles. It was previously shown that fusion of M2e to bacterial flagellin, the ligand for Toll-like receptor (TLR) 5 and powerful mucosal adjuvant, significantly increases the immunogenicity and protective capacity of M2e.

**Results:**

In this study, we report for the first time the transient expression in plants of a recombinant protein Flg-4M comprising flagellin of *Salmonella typhimurium* fused to four tandem copies of the M2e peptide. The chimeric construct was expressed in *Nicotiana benthamiana* plants using either the self-replicating potato virus X (PVX) based vector, pA7248AMV-GFP, or the cowpea mosaic virus (CPMV)-derived expression vector, pEAQ-*HT*. The highest expression level up to 30 % of total soluble protein (about 1 mg/g of fresh leaf tissue) was achieved with the PVX-based expression system. Intranasal immunization of mice with purified Flg-4M protein induced high levels of M2e-specific serum antibodies and provided protection against lethal challenge with influenza virus.

**Conclusions:**

This study confirms the usefulness of flagellin as a carrier of M2e and its relevance for the production of M2e-based candidate influenza vaccines in plants.

**Electronic supplementary material:**

The online version of this article (doi:10.1186/s12896-015-0164-6) contains supplementary material, which is available to authorized users.

## Background

Influenza is a widely distributed viral infection of humans and animals. The high variability of the surface viral proteins, hemagglutinin and neuraminidase, results in the appearance of a new epidemic strain every one to two years [1]; this requires the production of new vaccines at the same frequency. A promising solution to this problem is the development of recombinant vaccines that can be rapidly produced in standard expression systems. One of the most promising conserved antigens of influenza virus is the extracellular domain of transmembrane protein M2, M2e [2]. The length of M2e is only 24 amino acid residues (including the initiator methionine). Its sequence is virtually unchanged in all human isolates since 1933 [3–5], and in strains of avian influenza A it differs in only a few amino acids. By itself M2e possesses low immunogenicity [6] but this can be increased by delivery in a multimeric form or in combination with complex adjuvants or delivery systems [3, 5, 7–9].

Toll-like receptors (TLRs) have been shown to play a critical role in controlling the adaptive immune response, by properly arming dendritic and other antigen-presenting cells, triggering important costimulatory and regulatory mechanisms, and promoting antigen presentation [10, 11]. It has been previously shown that genetically fusing an antigen of interest to bacterial flagellin, the ligand for TLR5, significantly increases the immunogenicity and protective capacity of the antigen [12, 13]. Notably flagellin is particularly active as mucosal adjuvant, opening the possibility of non-invasive intranasal administration of vaccines. The ability of flagellin to be both the platform and the adjuvant for different vaccines was demonstrated in various models of infection including influenza [13–19].

Recombinant proteins, including those for medical purposes, can be produced in a variety of expression systems, including bacteria, yeast, plants, and mammalian cells. The advantages of using plants for protein production include the low final cost and inherent safety of products due to the absence of pathogens common for plants and animals. Recombinant proteins in plants may be obtained by stable genetic transformation (either nuclear or plastid) or through transient expression [20–22]. Creation of lines of stably transformed plants and, subsequently, production of a protein from such plants requires considerable time and cost. The level of expression of target proteins by transgenic plants is usually relatively low, resulting in a high cost of products due to the difficulty of their purification [23, 24]. The use of self-replicating viral vectors or efficient non-replicating transient expression vectors is an alternative approach providing sufficient amounts of the desired protein (up to 1–2 g/kg of plant biomass) [20] to conduct immunological studies. To create such vectors, sequences from tobacco mosaic virus (TMV), cowpea mosaic virus (CPMV) or potato virus X (PVX) have frequently been used. The genomes of these viruses are small and consist of positive-strand RNA. The vectors are introduced into plant cells by infiltration of the plant leaf tissue with *Agrobacterium* suspensions carrying the vector within the T-DNA portion of a binary plasmid resulting in transfer of vector DNA into the recipient plant cells during the agroinfection [25, 26]. Such expression systems have been used for production of a variety of pharmaceutical proteins, including antibodies and candidate vaccines [22, 27, 28]. The latter have included influenza virus hemagglutinin [29–31] and HBc virus-like particles carrying M2e peptide [9].

In this work four tandem copies of M2e sequences based on human and avian influenza virus isolates were genetically fused to *Salmonella typhimurium* flagellin and produced in *Nicotiana benthamiana* plants using transient expression using either a replicating PVX vector [32] or the non-replicating CPMV-*HT* system [33, 34]. Intranasal immunization of mice with the plant-produced hybrid protein induced the desired immune response and conferred protective immunity against a lethal influenza virus challenge.

## Results

### Viral vectors for expression of the hybrid protein consisting of flagellin linked to four copies of influenza M2e peptide in N. benthamiana plants

**Fig. 1:**
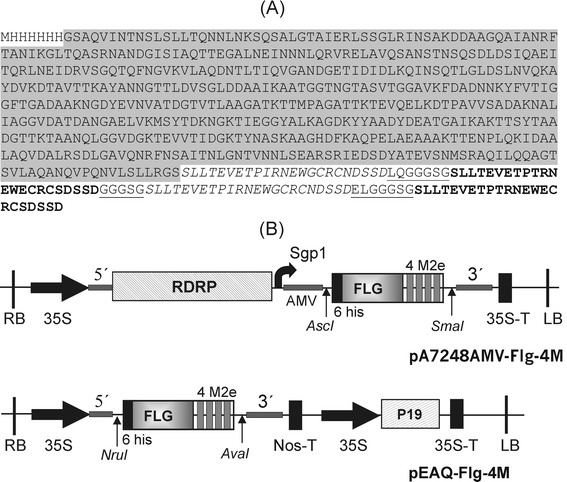
Structure of the expression constructs used in this study. **a** Amino acid sequence of Flg-4M protein. The sequences of human “consensus” M2e peptides, M2eh, are italicized; the sequences of M2e peptide of avian influenza virus strain A/Chicken/Kurgan/05/2005, M2ek, are shown in bold; the linker sequences are underlined. Flagellin sequence is shaded in grey. **b** Structures of viral vectors pA7248AMV-Flg-4M and pEAQ-Flg-4M. The tDNA regions of the binary vectors are shown. RDRP, RNA dependent RNA polymerase of PVX; Sgp1, subgenomic promoter of the PVX protein 25 K; AMV, the leader sequence of RNA 4 of the alfalfa mosaic virus; 6his, 6 histidine tag; FLG, flagellin of *S. typhimurium*; 4M2e, four copies of M2e peptide (arranged as M2eh-M2ek-M2eh-M2ek); 35S, promoter; 35S-T, terminator of the cauliflower mosaic virus RNA; NosT, terminator of nopaline synthase gene; LB and RB are the left and right borders of tDNA; p19, gene of silencing suppressor P19 from the tomato bushy stunt virus; 5’ and 3’, the 5’-UTR and the 3’-UTR from PVX (in pA7248AMV-Flg-4M) and CPMV (in pEAQ-Flg-4M)

The highly conserved extracellular domain of the influenza virus M2 protein, M2e, is a promising target for the development of recombinant “universal” vaccines against influenza [5]. However, M2e is poorly immunogenic and needs to be presented in a multimeric form or linked to a carrier molecule such as virus-like particles [3]. Here we use flagellin of *Salmonella typhimurium* as such carrier and simultaneously as an adjuvant. In order to make a broader range candidate vaccine, a hybrid protein Flg-4M containing two copies of human consensus M2e sequence and two copies of the M2e peptide of avian influenza virus strain A/Chicken/Kurgan/05/2005 fused to the C-terminus of *S. typhimurium* FljB (arranged as Flg-M2eh-M2ek-M2eh-M2ek) was designed. In order to facilitate folding of hybrid protein, copies of M2e peptides were separated from each other by flexible glycine-rich linker (Fig. 1a). The empty flagellin (lacking M2e) was used as a control. Both proteins, Flg-4M and Flg, were designed with N-terminal 6-histidine tag to facilitate their purification.

Two plant virus-based transient expression systems were used for production of recombinant Flg-4M protein in *N. benthamiana*. The first vector, pA7248AMV-GFP [32], is a replicating system based on the PVX genome. This vector includes the 5’-UTR of the PVX genome, the polymerase gene, the first promoter of subgenomic RNA, the translation enhancer represented by the sequence of the 5’-untranslated region of RNA 4 of the alfalfa mosaic virus (AMV), the GFP gene flanked by unique restriction sites *Asc*I and *Sma*I, the last 60 nucleotides of the coat protein gene, and the 3’-untranslated region of the PVX genome. This construction is nserted between the 35S promoter and 35S terminator and cloned in the binary vector pBIN19 [32]. To create the viral vector allowing production of the Flg-4M protein, a sequence of the hybrid gene was cloned in pA7248AMV-GFP at the *Asc*I and *Sma*I sites to replace GFP, which resulted in construction of a recombinant vector pA7248AMV-Flg-4M (Fig. 1b). Recombinant vector pA7248AMV-Flg for expression of Flg in plants was constructed in a similar way.

The second expression system, pEAQ, is based on CPMV. This is a non-replicating system which uses production of a highly translatable mRNA to achieve high level expression. The recombinant vector pEAQ-Flg-4M contains 5’-UTR and 3’-UTR from CPMV RNA-2 upstream and downstream, respectively, of the hybrid gene Flg-4M (Fig. 1b).

### Expression of the hybrid protein Flg-4M in N. benthamiana plants

**Fig. 2:**
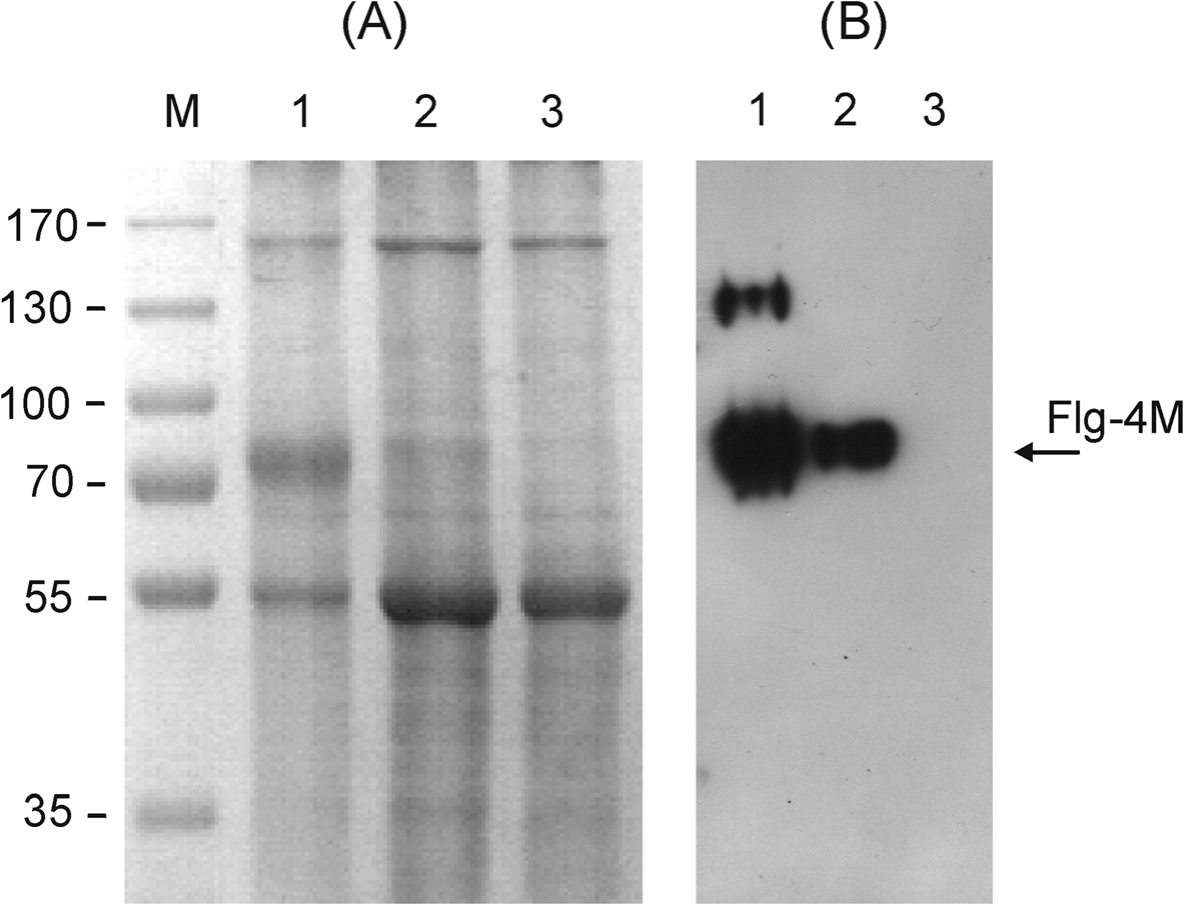
Efficiency of PVX- and CPMV-based vectors. Coomassie brilliant blue stained gel (**a**) and western blot (**b**) of proteins isolated form *N. benthamiana* plants and separated by SDS-PAGE. M, − molecular weight marker (kD); 1 and 2, − total soluble proteins isolated at fourth day after agroinfiltration from leaves infiltrated with vector pA7248AMV-Flg-4M and pEAQ-Flg-4M, respectively; 3, − total soluble proteins isolated from empty leaves. Antibodies against M2e were used in western blotting

To express the protein Flg-4M, vectors were inserted into *A. tumefaciens* strain GV3101, which was used for agroinfiltration of leaves of *N. benthamiana* plants. Agroinfiltration zones for two vectors were located within one leaf. Protein samples were isolated in 3, 4, 5, 6, and 7 days after infection. For every day three leaves were taken for analysis. Protein samples were analyzed using SDS-PAGE and western-blotting (Fig. 2). Maximal expression was observed at fourth day. Summarizing the expression results for 15 leaves we found out that pEAQ-Flg-4M construct provides expression level of about 30 % in comparison to pA7248AMV-Flg-4M.

**Fig. 3:**
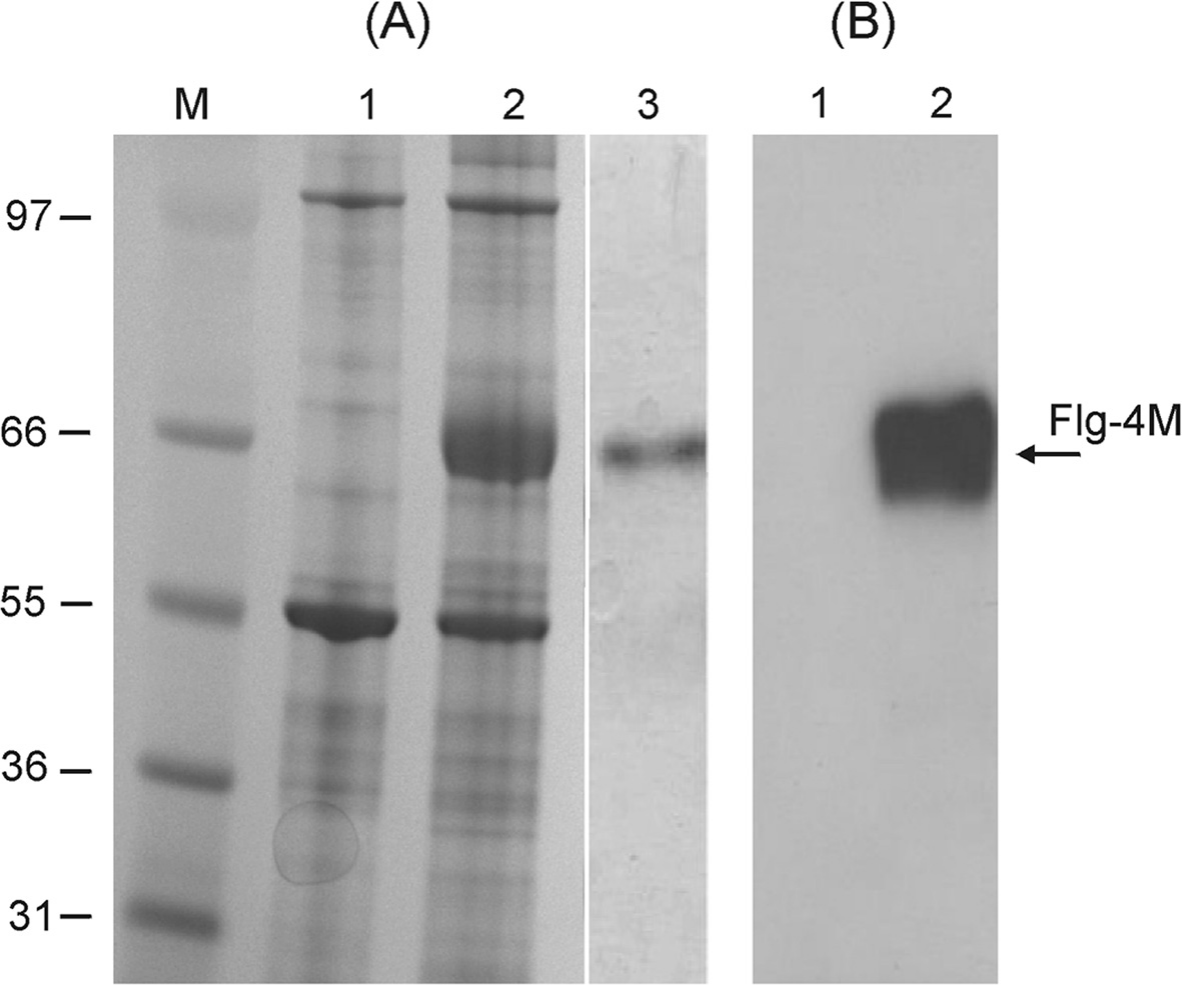
Expression and purification of Flg-4M protein. Coomassie brilliant blue stained gel (**a**) and western blot (**b**) of proteins isolated form *N. benthamiana* plants and separated by SDS-PAGE. M, − molecular weight marker (kD); 1, − total soluble proteins isolated from empty leaves; 2, − total soluble proteins isolated at fourth day after agroinfiltration from leaves infiltrated with vector pA7248AMV-Flg-4M; 3, − purified Flg-4M protein. Antibodies against M2e were used in western blotting

Given the higher levels of expression obtained, we used vector pA7248AMV-Flg-4M for the scaled-up production of protein Flg-4M in *N. benthamiana* plants. The total soluble protein was isolated from leaves at 4th day after agroinfiltration and analyzed by SDS-PAGE. The data presented in Fig. 3a show that Flg-4M protein is highly expressed and accounted for about 30 % of the soluble protein fraction (~1 mg/g of fresh leaf tissue). The recombinant protein was specifically revealed in western blot analysis with the monoclonal antibodies specific to M2e (Fig. 3b). The recombinant fusion protein carrying an N-terminal 6-histidine tag was purified to a purity of greater than 90 % (Fig. 3a) using standard affinity chromatography on Ni-NTA resin and used for animal experiments. The recombinant Flg protein was expressed in *N. benthamiana* using vector pA7248AMV-Flg and purified in a similar way.

### Antigenicity of plant-produced protein Flg-4M

**Fig. 4:**
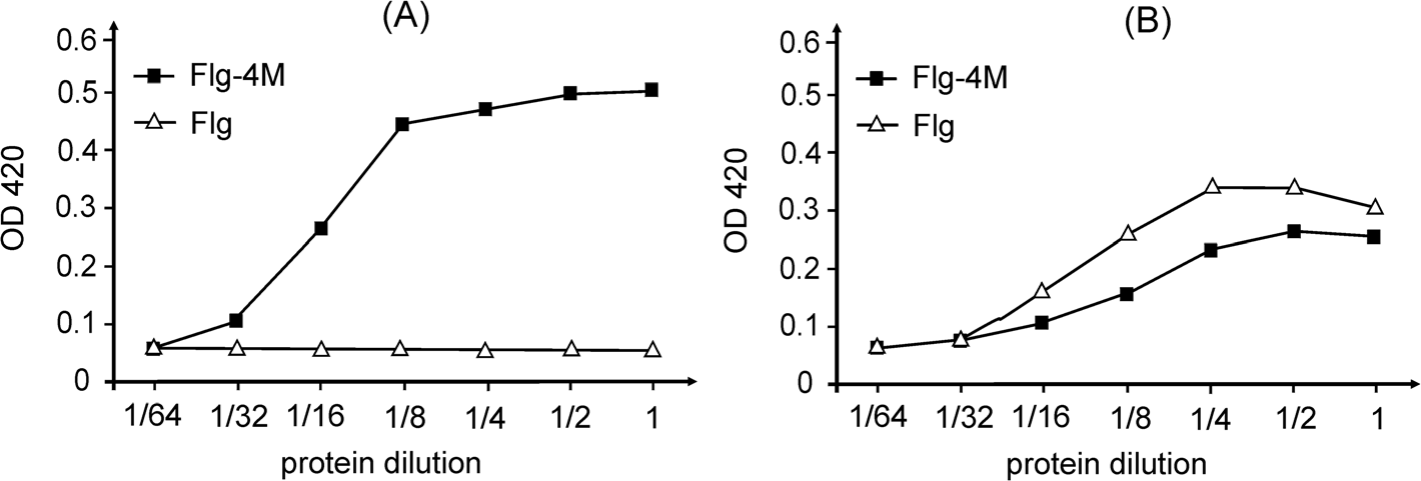
Antigenicity of Flg-4M protein. Two-fold dilutions of recombinant Flg-4M or Flg proteins (starting from 30 μg/ml) were coated on ELISA plates which were then probed with monoclonal antibodies specific for M2e (**a**) or 6-his tag present both in Flg-4M and Flg (**b**)

The display of the M2e peptide in an accessible manner when it is fused with flagellin in the Flg-4M protein was examined by ELISA. To this end plates were coated with serial dilutions of Flg-4M protein and empty Flg, then probed with the M2e-specific and the 6-his tag-specific monoclonal antibodies (Fig. 4). While both Flg-4M and Flg reacted with the anti-his antibodies, only Flg-4M reacted with the M2e-specific antibodies. These results confirm the presence of M2e in the purified Flg-4M protein and its accessibility for antibodies.

The M2e peptides naturally exposed on the surface of infected cells as tetramers tends to aggregate due to the presence of two cysteine residues per M2e copy that lead to disulfide bond formation and intra- and interprotein cross-linking. In order to control the lack of aggregation of Flg-4M protein we also performed western-blot analysis of purified protein under reducing and non-reducing conditions. The recombinant protein migrated at expected position and was revealed by the monoclonal antibodies specific to M2e, suggesting the lack of aggregation (see Additional file 1). No high-molecular weight aggregates or particulate structures were detected by the electron microscopy of purified protein samples.

### Immunogenicity and protectivity of plant-produced protein Flg-4M

**Fig. 5:**
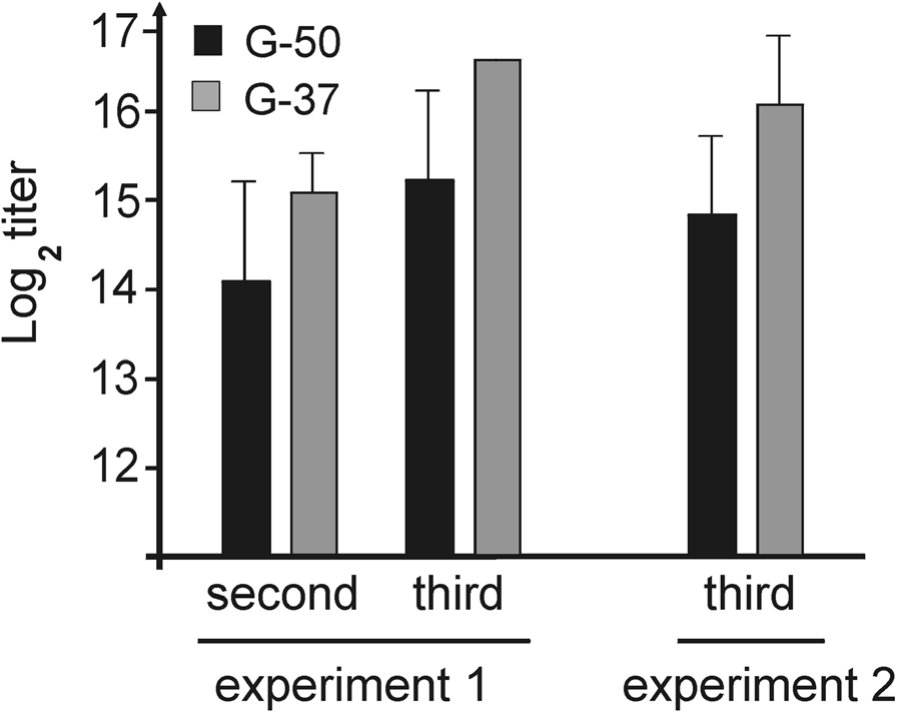
Titers of IgG antibodies to synthetic M2e peptides G-50 and G-37 in sera of immunized mice. The data obtained in two independent experiments after the second and the third immunizations are shown. The results are expressed as the mean titer ± standard deviation for each group of five mice. Titers lower than 200 were detected for mice immunized with PBS or Flg

To characterize the immunogenicity and protective action, we performed two sets of animal experiments. In the first case mice were immunized with the plant-produced preparation of Flg-4M protein, in the second experiment an additional group of mice was immunised with plant-produced flagellin lacking M2e. Mice were vaccinated intranasally three times at two-week intervals with 10 μg protein without additional adjuvants. Blood samples were taken 2 weeks after the second and/or third immunizations. Serum was analysed by ELISA to identify IgG antibodies directed against M2e, using plates coated with synthetic peptides G-37 and G-50, whose sequences corresponded to the human “consensus” M2e sequence and the M2e of influenza strain A/Chicken/Kurgan/05/2005, respectively. A strong immune response was activated after the second immunisation and further increased after the third (Fig. 5). Induced antibodies efficiently bind to both synthetic M2e peptides G-50 and G-37, which are present in the candidate vaccine, although the immune response towards G37, the human M2e sequence, was stronger.

**Fig. 6:**
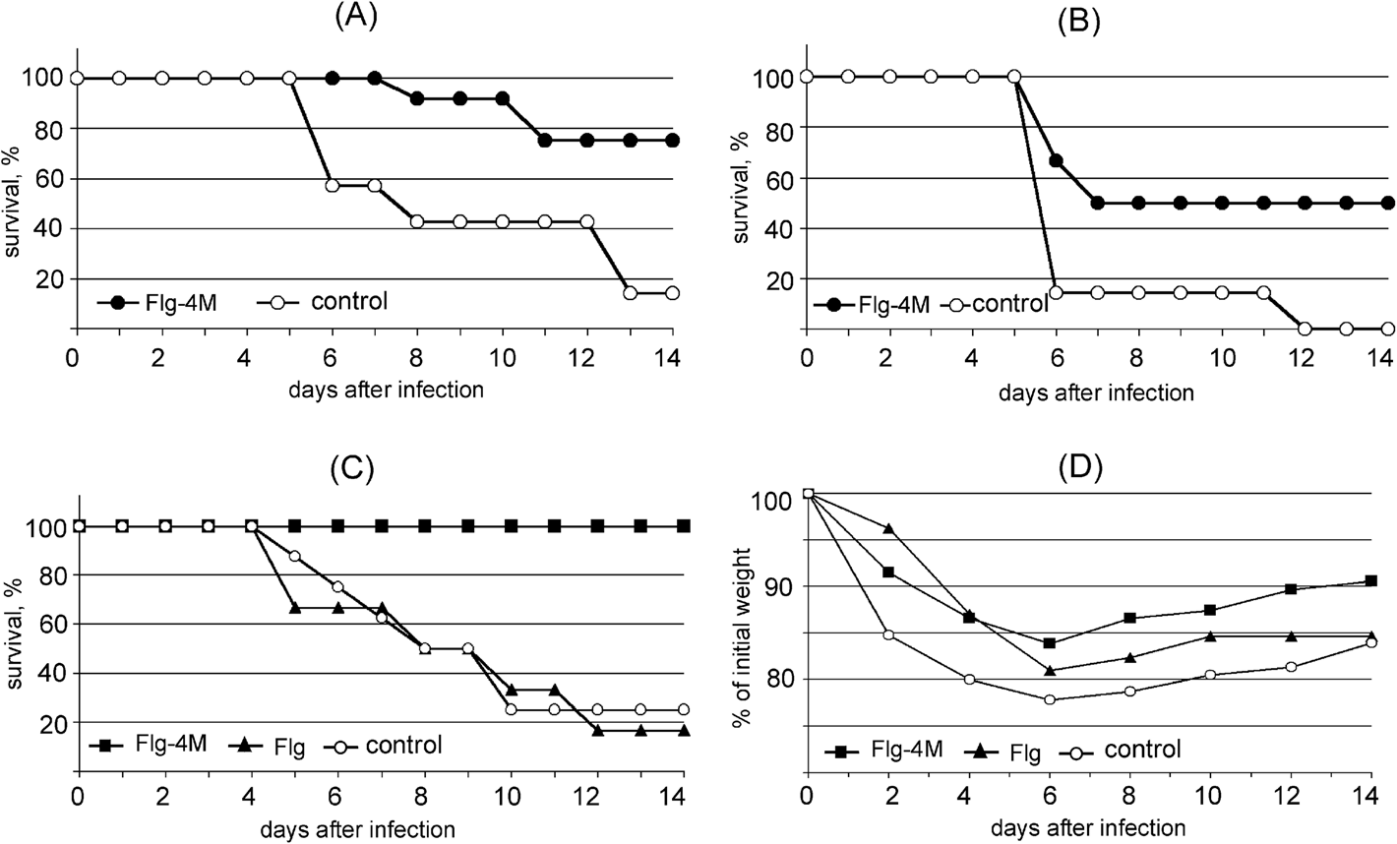
Protective efficiency of the plant-produced Flg-4M protein. Experiment 1: mice were challenged with 5LD_50_ of A/Chicken/Kurgan/05/2005 (**a**) or with 10LD_50_ of A/PR/8/34 (**b**). Experiment 2: mice were challenged with 5LD_50_ of influenza strain A/PR/8/34 (**c, d**). Survival (**a, b, c**) and weight (**d**) of immunized and control mice were monitored for 14 days post-challenge. Control, mice immunized with PBS

In the first experiment the immunised and control mice were challenged with 10LD_50_ of mouse-adapted human influenza strain A/PR/8/34 (H1N1) and with 5LD_50_ of avian influenza strain A/Chicken/Kurgan/05/2005 (Н5N1). As shown in Fig. 6a, 75 % mice immunized with Flg-4M, survived the lethal challenge of A/Chicken/Kurgan/05/2005 (Н5N1), while the rate of survival among the control mice was 14 %. The challenge of immunized mice with 10LD_50_ of A/PR/8/34 (H1N1) led to a 50 % survival, while all control mice died upon infection (Fig. 6b).

In the second experiment 5LD_50_ of influenza strain A/PR/8/34 (H1N1) was used for challenge. Figure 6c shows that mice immunised with Flg-4M were completely protected from lethal virus challenge, while mice immunised with Flg or PBS were not protected. The morbidity of the disease was monitored by measuring the weight of the animals. The data presented in Fig. 6d reveal that immunisation did not eliminate morbidity but reduced it relative to the control.

## Discussion

The purpose of the present work was to develop a plant-produced candidate influenza vaccine based on M2e peptide linked to bacterial flagellin acting as an adjuvant for mucosal immunization. It was previously demonstrated [13] that immunization of mice with *E. coli*-produced flagellin linked to four tandem copies of human “consensus” M2e, provides protection against lethal challenge with human influenza A strain. Although the M2e sequence is well conserved in almost all influenza strains isolated from humans, the sequences of M2e peptides of newly emerging highly pathogenic strains of animal origin, such as avian influenza, differ from the human consensus, suggesting that a specific M2e-based vaccine against these strains would be required [35]. Combination of several M2e peptides of different origin in one vaccine protein could allow development of candidate vaccine efficient against the corresponding strains [36]. Therefore in addition to human consensus M2e peptide we included M2e peptides of avian influenza virus into the hybrid protein based on bacterial flagellin.

Although plants for a long time have been recognized as a promising host for expression of recombinant proteins, including vaccines, there are no universal approaches ensuring high level expression for any protein of interest. Here we used two plant viral-based expression system for production of Flg-4M protein in *N. benthamiana*. The first is replicating vector pA7248AMV-GFP based on PVX genome, and the second vector pEAQ comprising fragments of CPMV genome is unable to replicate in plant cell. In this instance the replicating PVX-based vector gave higher expression of the Flg-4M protein than pEAQ. The results obtained here are in contrast to those found when comparing the expression of the Human Papillomavirus 8 L1 protein using a replicating TMV-based vector and the pEAQ-*HT* system where the latter gave a 15-fold higher level [37]. There are several possible explanations for this difference in expression levels between the two systems: it may reflect an intrinsic higher level of expression from the replicating versus the non-replicating system or it may relate to the strain of *A. tumefaciens* (GV3101 vs LBA4404) used for infiltration. The target protein was expressed in *N. benthamiana* cells at very high level up to 30 % of total soluble protein for pA7248AMV-GFP (~1 mg/g of fresh leaf tissue). It is much higher than 280 μg/g of fresh weight leaves reported for expression of the flagellin alone using non-replicating transient expression system [38]. Analysis of the plant-expressed Flg-4M protein by transmission electron microscopy did not reveal the presence of aggregates. This coupled with observation that the protein remained in the supernatant after ultracentrifugation suggests that the protein remains in an essentially unaggregated state.

Intranasal immunization of mice with the plant-produced Flg-4M protein induced an immune response against M2e and was responsible for development of protective immunity against the lethal influenza infection. Complete protection was observed in an experiment where immunized mice were challenged with 5LD_50_ of human influenza strain A/PR/8/34. Notably the protection strongly depended on the presence of M2e in the hybrid protein, since immunisation with plant-produced empty Flg confer no extra protection relative to the negative control. In other experiments the protection was, however, not 100 % complete that could be related to low immunization dose (only 10 μg) and, in case of experiment with 10LD_50_ of A/PR/8/34 strain, by the use of too high infection dose, as evidenced by complete mortality in the control group.

The lack of complete protection of mice was observed by Stepanova *et al.* [39] in case of Flg-4M protein produced in *E. coli*: 90 % survival upon challenge with 5LD_50_ of A/Chicken/Kurgan/05/2005 and 80 % survival upon challenge with 5LD_50_ of A/PR/8/34 strain, although a higher dose of vaccine protein, 50 μg, was used for each of three immunisation.

Three other examples of the plant-based expression of the M2e epitope have been reported. Ravin *et al.* [9], using the PVX-based vector employed in this work, produced HBc virus-like particles carrying the M2e peptide with the yield about 1-2 % of total soluble protein; the plant-produced particles induced a protective immune response (90 % survival) against 1LD_50_ of homologous influenza A infection. Virus-like particles formed by L1 protein of human papillomavirus fused to M2e were produced in *N. benthamiana* with the pEAQ-*HT* expression system with the yield in the range from 45 to 120 μg/g of fresh weight [40]. Another approach is construction of recombinant tobacco mosaic virus carrying M2e epitope as a fusion with the coat protein; such viruses are capable of systemic infection of *N. benthamiana* plants and induced a protective immune response against challenge with 5LD_50_ of homologous influenza A virus [41]. Altogether these results indicated that production of M2e-based candidate influenza vaccines in plants is feasible. The particular advantage of flagellin as a mucosal adjuvant suggests that expression of flagellin-M2e hybrid protein in plants by agroinfiltration with a recombinant viral vectors is a promising approach for production of recombinant vaccines against influenza.

## Conclusions

In this study, we expressed the chimeric protein comprising flagellin of *Salmonella typhimurium* fused to four tandem copies of the influenza M2e peptide in *N. benthamiana* plants. The use of self-replicating viral vector based on PVX genome allowed to achieve very high level of expression up to 1 mg/g of fresh leaf tissue. Intranasal immunization of mice with purified vaccine protein induced high levels of M2e-specific serum antibodies and provided protection against lethal challenge with influenza virus. This work shows that expression of fusion proteins based on flagellin and M2e in plants is feasible and could became a promising approach for manufacturing of “universal” recombinant vaccines against influenza.

## Methods

### Media, reagents and synthetic gene

Bacteria were grown in LB broth or on plates with LB agar at 37 °C (*Escherichia coli*) or at 28 °C (*Agrobacterium tumefaciens*). If necessary, the media were supplemented with antibiotics: kanamycin (50 μg/ml), rifampicin (50 μg/ml), or gentamycin (25 μg/ml). Monoclonal antibodies to the M2e (clone M2A10) were generously provided by Dr. Peter Sveshnikov from the Russian Research Center of Molecular Diagnostics and Therapy.

Previously we constructed an artificial gene Flg-4M encoding a hybrid protein carrying an N-terminal histidine tag and consisting of flagellin of *Salmonella typhimurium* (Flg) with four copies of M2e peptide linked to its C terminus [39]. The M2e sequences corresponded to a human “consensus” M2e peptide (SLLTEVETPIRNEWGCRCNDSSDP, M2eh), and the M2e peptide of avian influenza virus strain A/Chicken/Kurgan/05/2005 (SLLTEVETPTRNEWECRCSDSSD, M2ek). Individual copies of the M2e peptides were separated from each other by flexible glycine-rich linkers. The hybrid gene, arranged as Flg-M2eh-M2ek-M2eh-M2ek, was cloned in expression vector pQE30 resulting in plasmid pQE30_Flg2M2eh2M2ek. The amino acid sequence of the hybrid protein Flg-4M is shown in Fig. 1a.

### Vectors for expression of target proteins in plants

For expression of the hybrid protein Flg-4M in plants the corresponding gene was cloned into vectors pA7248AMV-GFP (based on the PVX genome [32]) and pEAQspecialK-GFP-HT (a non-replicating CPMV-based vector [34]). These are the binary vectors and can be maintained in both *E. coli* and *A. tumefaciens*. The sequence encoding Flg-4M was amplified by PCR using primers AscI-Flg-F (ATGGCGCGCCATGCATCACCATCACCATCACGGA) and M2K-SmaI-R (ATCCCGGGAATTAAGCTGGGGTACCCTAATCG) or NruI-Flg-F (ATTCGCGAATGCATCACCATCACCATCACGGA) and M2K-AvaI-R (ATCTCGAGAATTAAGCTGGGGTACCCTAATCG) using pQE30_Flg2M2eh2M2ek as a template for PCR-amplification.

For expression of flagellin lacking M2e (Flg) the corresponding gene was amplified by PCR using primers AscI-Flg-F (ATGGCGCGCCATGCATCACCATCACCATCACGGA) and Flg-SmaI-R (ATCCCGGGCTAACGTAACAGAGACAGCACGTTC) and pQE30_Flg2M2eh2M2ek as a template.

The PCR fragments encoding Flg-4M were cloned in pA7248AMV-GFP at the *Asc*I/*Sma*I sites or in pEAQspecialK-GFP-HT at the *Nru*I/*Ava*I sites. The resulting recombinant vectors, pA7248AMV-Flg-4M and pEAQ-Flg-4M (Fig. 1b), were transferred from *E. coli* into the *A. tumefaciens* strain GV3101 using electroporation. Likewise the PCR fragment encoding Flg was cloned in pA7248AMV-GFP at the *Asc*I/*Sma*I sites resulting in recombinant vector pA7248AMV-Flg for expression of 6-his tagged flagellin in plants.

### Agroinfiltration of plants

Agrobacteria carrying recombinant binary vectors were grown overnight with shaking at 28 °C. The cells (1.5 ml) were harvested by centrifugation for 5 min at 4000 g, and the pellet was resuspended in 1.5 ml of buffer containing 10 mM MES (pH 5.5) and 10 mM MgSO_4_. Leaves of *N. benthamiana* plants were infiltrated with suspension of agrobacteria (optical absorption of the solution OD_600_ ~ 0.2) using a syringe without a needle.

For expression of pA7248AMV-Flg-4M, the plant leaves were concurrently infiltrated with agrobacteria carrying binary vector driving expression of silencing suppressor 24-kDa protein (p24) from grapevine leafroll-associated virus-2 [42] to suppress virus-induced gene silencing. The same protocol was used for expression of Flg.

### Isolation of proteins from plant tissues

The plant-produced Flg-4M protein carrying 6-histidine tag was isolated under denaturing conditions on Ni-NTA Resin (Promega, USA). Four to five days after infiltration, the *N. benthamiana* leaves were powdered to homogeneity in liquid nitrogen. A solution containing 6 M Guanidine HCI, 0.1 M NaH_2_PO_4_, 0.01 M Tris (pH 8.0) was added to the powdered plant tissue (5 mL per 1 g). The resulting mixture was centrifuged at 14,000 g for 15 min, and the supernatant was applied to Ni-NTA-resin equilibrated with the same buffer and incubated for 60 min. Then the resin was sequentially washed with PBS (50 mM NaH_2_PO_4_, 300 mM NaCI, pH 8.0) buffer containing 10 mM and 20 mM imidazole, respectively. The recombinant protein Flg-4M was eluted with PBS containing 0.5 M imidazole. After elution the protein was dialyzed against PBS using Slide-A-Lyzer Mini dialysis devices (Thermo Scientific, USA). Dialyzed protein preparation was centrifuged at 100,000 g to remove Rubisco aggregates and then supernatant was stored at −20 °C in PBS. Total soluble proteins were measured by a Bradford assay (Bio-Rad) following manufacturer’s instructions. The same protocol was used for purification of Flg.

### SDS-PAGE and western-blotting of protein preparations

Protein samples were diluted in SDS-PAGE sample buffer including β-mercaptoethanol. The samples were boiled for 5 min and subjected to SDS-PAGE in a 10 % (w/v) gel. After electrophoresis, the gel was either stained with Coomassie brilliant blue or the proteins were transferred from the gel onto a Hybond-P membrane (GE Healthcare, USA) by electroblotting. To prevent nonspecific binding of antibodies with the membrane, it was treated with 5 % (w/v) solution of dry milk in TBS-T (150 mM NaCI, 20 mM Tris, 0.1 % Tween 20, pH 8.0) buffer. The membrane was probed with mouse monoclonal antibodies specific for the M2e peptide and then incubated with rabbit anti-mouse antibodies conjugated with peroxidase. Specific protein complexes were detected using a Western Blot ECL Plus kit (GE Healthcare, USA). The intensity of the bands in stained gels and blots was determined using Nonlinear. Dynamics. TotalLab. TL120.v2009-NULL software.

Western blot analysis of purified Flg-4M under non-reducing conditions was performed as above but that β-mercaptoethanol was not included in the sample buffer and the sample was not boiled before loading onto a gel.

### Protein ELISA

To confirm identity and accessibility of M2e to antibodies, ELISA plates were coated overnight at 4 °C with serial dilutions of Flg-4M or Flg proteins in sodium bicarbonate buffer pH 8.5. Plates were treated with a blocking buffer supplemented with 0.1 % (w/v) BSA for 2 h at 37 °C and washed three times using PBS with Tween. Plates were probed with monoclonal antibody specific for M2e or 6-His tag for 1 h at 37 °C. As a conjugate, rat monoclonal anti-mouse IgG (Imtek, Russia) labelled with a horseradish peroxidase was used at a 1:10,000 dilution. After adding tetramethylbenzidine substrate (BD Bioscience) and monitoring color development, the reaction was stopped by H_2_SO_4_, and OD at 450 nm was measured on a microplate spectrophotometer.

### Synthetic peptides

The following synthetic peptides were tested as a positive control in the ELISA experiments:

G-50 SLLTEVETPTRNEWECRCSDSSD (M2ek, A/Chicken/Kurgan/05/2005)

G-37 SLLTEVETPIRNEWGCRCNDSSD (M2eh, human consensus). Residues that differ between the sequences are underlined.

### Analysis of production of antibodies against M2e

Female Balb/c mice weighing 18–20 g were immunized intranasally thrice at two week intervals. The dose of Flg-4M and Flg proteins was 10 μg/mouse and no additional adjuvant was used. The mouse sera were studied two weeks after the second and third immunizations. The sera of non-immunized mice were used as a negative control.

For ELISA, 96-well plates with a high adsorption capacity (Greiner, Germany) were covered with synthetic peptides G-50 (M2ek) and G-37 (M2eh) at a concentration of 5 mg/ml (w/v) in phosphate buffer, pH 7.2–7.4 and kept overnight at 4 °C. Plates were treated with a blocking buffer (0.01 M PBS, pH 7.2–7.4) supplemented with 5 % (v/v) fetal calf serum for 1 h at room temperature and washed three times using PBS with Tween. A series of 1:2 dilutions of the different serum samples, starting with a 1:400 dilution, were loaded onto the antigen-coated plates in the blocking buffer and then incubated for 1 h at room temperature. As a conjugate, rat monoclonal anti-mouse IgG (Invitrogen) labelled with a horseradish peroxidase was used at a 1:2000 dilution. Tetramethylbenzidine (BD Bioscience) was used as a substrate. The reaction was monitored by measuring the OD at 450 nm. The endpoint titres are defined as the highest dilution producing an OD value twice that of the background (serum of non-immunised mice).

### Influenza viruses and challenge

The following mouse-adapted influenza viruses were used to challenge animals immunised by the candidate vaccines: A/PR/8/34 (H1N1) at the dose of 10LD_50_ or 5LD_50_, and A/Chicken/Kurgan/05/2005 (Н5N1) at the dose of 5LD_50_. The virus was administered intranasally in a total volume of 100 μl to mice anaesthetised by ether. The mice were monitored for weight loss and survival rate daily following the viral challenge for a period of 2 weeks. Survival rate was determined by death or a cut-off of 25 % in body weight loss at which point animals were euthanized.

### Ethics statement

The study was carried out in accordance with the Russian Guidelines for the Care and Use of Laboratory Animals. The protocol was approved by the Committee for Ethics of Animal Experimentation of the Research Institute of Influenza (Permit Number: 01213). All efforts were made to minimize animal suffering.

## Additional file

Additional file 1: Figure S1. Western- blot analysis of purified Flg-4M protein under reducing and nonreducing conditions.

## Abbreviations

AMV: 5’-untranslated region of RNA 4 of the alfalfa mosaic virus
CPMV: Cowpea mosaic virus
M2e: The extracellular domain of matrix protein 2 of influenza A virus
PVX: Potato virus X
TLR: Toll-like receptor
TMV: Tobacco mosaic virus
