# Supplementary material for: Rapid high-yield expression of a candidate influenza vaccine based on the ectodomain of M2 protein linked to flagellin in plants using viral vectors
Source: BMC Biotechnol. 2015 May 29;15:42. doi: 10.1186/s12896-015-0164-6 (PMC4446962; doi:10.1186/s12896-015-0164-6)
Supplement: Supplementary file 1 — Western- blot analysis of purified Flg-4M protein under reducing and nonreducing conditions. [file 12896_2015_164_MOESM1_ESM.pdf]

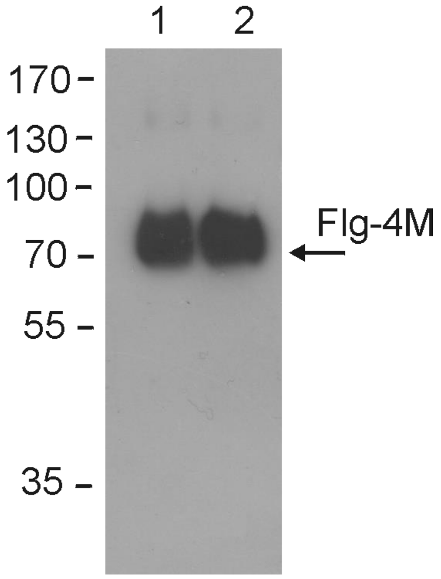

Figure S1 Western- blot analysis of purified Flg-4M protein under reducing and non-reducing conditions.
1, - purified Flg-4M protein analysed by SDS-PAGE under non-reducing conditions;
2, - purified Flg-4M protein analysed by SDS-PAGE under reducing conditions.
Positions of molecular weight marker (kD) are shown on the left. Antibodies against M2e were used in Western blotting.
